# A Methodological Framework for the Reconstruction of Contiguous Regions of Ancestral Genomes and Its Application to Mammalian Genomes

**DOI:** 10.1371/journal.pcbi.1000234

**Published:** 2008-11-28

**Authors:** Cedric Chauve, Eric Tannier

**Affiliations:** 1 Department of Mathematics, Simon Fraser University, Burnaby, British Columbia, Canada; 2 INRIA, Rhône-Alpes, France; 3 Université de Lyon, Lyon, France; 4 Université Lyon 1, Lyon, France; 5 Laboratoire de Biométrie et Biologie Évolutive, CNRS, UMR5558, Villeurbanne, France; UCSD, United States of America

## Abstract

The reconstruction of ancestral genome architectures and gene orders from homologies between extant species is a long-standing problem, considered by both cytogeneticists and bioinformaticians. A comparison of the two approaches was recently investigated and discussed in a series of papers, sometimes with diverging points of view regarding the performance of these two approaches. We describe a general methodological framework for reconstructing ancestral genome segments from conserved syntenies in extant genomes. We show that this problem, from a computational point of view, is naturally related to physical mapping of chromosomes and benefits from using combinatorial tools developed in this scope. We develop this framework into a new reconstruction method considering conserved gene clusters with similar gene content, mimicking principles used in most cytogenetic studies, although on a different kind of data. We implement and apply it to datasets of mammalian genomes. We perform intensive theoretical and experimental comparisons with other bioinformatics methods for ancestral genome segments reconstruction. We show that the method that we propose is stable and reliable: it gives convergent results using several kinds of data at different levels of resolution, and all predicted ancestral regions are well supported. The results come eventually very close to cytogenetics studies. It suggests that the comparison of methods for ancestral genome reconstruction should include the algorithmic aspects of the methods as well as the disciplinary differences in data aquisition.

## Author Summary

No DNA molecule is preserved after a few hundred thousand years, so inferring the DNA sequence organization of ancient living organisms beyond several million years can only be achieved by computational estimations, using the similarities and differences between chromosomes of extant species. This is the scope of “paleogenomics”, and it can help to better understand how genomes have evolved until today. We propose here a computational framework to estimate contiguous segments of ancestral chromosomes, based on techniques of physical mapping that are used to infer chromosome maps of extant species when their genome is not sequenced. This framework is not guided by possible evolutionary events such as rearrangements but only proposes ancestral genome architectures. We developed a method following this framework and applied it to mammalian genomes. We inferred ancestral chromosomal regions that are stable and well supported at different levels of resolution. These ancestral chromosomal regions agree with previous cytogenetics studies and were very probably part of the genome of the common ancestor of humans, macaca, mice, dogs, and cows, living 120 million years ago. We illustrate, through comparison with other bioinformatics methods, the importance of a formal methodological background when comparing ancestral genome architecture proposals obtained from different methods.

## Introduction

The reconstruction of ancestral karyotypes and gene orders from homologies between extant species is a long-standing problem [1]. In the case of mammalian genomes, it has first been approached using cytogenetics methods [2]–[7]. The recent availability of sequenced and assembled genomes has led to the development of bioinformatics methods that address this problem at a much higher resolution, although with fewer available genomes. Such methods propose in general more detailed ancestral genome architectures than cytogenetics methods (see [8]–[12] and reviews in [13]–[15]). The comparison of the two approaches was recently investigated and discussed in a series of papers, sometimes with diverging point of views [16]–[18]. Among the bioinformatics methods that have been applied to mammalian genomes (previous works were limited to small genomes such as organellar genomes [19] or to bacterial genomes [20]), the one based on a parsimony approach in terms of evolutionary events such as reversals, translocations, fusions and fissions [8],[11], leads to results that are sometimes in disagreement with cytogenetics studies [16]. Recent results on this approach point out that the modeling of genome rearrangements probably needs further studies before it can be used for the reconstruction of ancestral genomes (see [21], or [17], where it was suggested that inferring parsimonious rearrangement scenarios is more intended to infer evolutionary dynamics characteristics, such as rearrangement rates, than ancestral genomes). Another type of approach infers ancestral genome segments, called Contiguous Ancestral Regions (CARs), from syntenic features that are conserved in extant species (the terminology is borrowed from [12]). We call this principle *model-free*, following [22], even if it is based on certain assumptions, like the absence of events inside a conserved synteny, which is a parsimony principle. But this terminology stresses the difference with rearrangement-based methods, which contraint the reconstruction by allowing prescribed operations that define then an evolution model. It is then less ambitious than the rearrangement-based approach as it does not propose evolutionary events, neither does it ensure that proposed CARs are ancestral whole chromosomes. However, when recently applied on mammalian genomes [12] it gave results more in agreement with cytogenetic methods, while exhibiting few other points of divergence [18].

We describe here a very general model-free framework for the reconstruction of CARs, that formalizes and generalizes the principles used in several computational [12],[22] and cytogenetics [5]–[7] studies. This framework takes as input a representation of extant genomes as sequences of homologous genomic markers (synteny blocks or orthologous genes for example). Then it decomposes into two main steps: we first compute a collection of possible ancestral syntenic groups (in general small groups of genomic markers that were possibly contiguous in the ancestral genome), each weighted according to its conservation in the extant species; from this set of possible ancestral syntenies, we group and order the considered genomic markers into one (or several alternative) set(s) of CARs, each of these sets of CARs representing a possible ancestral genome architecture. An important feature of our framework is that we propose the set of all possible genome architectures that agree with the conserved ancestral syntenies. This framework is general in the sense that both steps can be made effective in several ways. For example, during the first phase, the signal for ancestral syntenies can be defined from extant species in terms of conserved adjacencies between homologous markers as in [12] or between chromosome segments as in [5]–[7]. We propose one possible implementation of this framework, choosing as ancestral features both conserved adjacencies and gene teams [23],[24], generalizing the approach of Ma *et al.* [12] (where only adjacencies were considered), and mimicking the methods employed with cytogenetic data [5]–[7] (conserved chromosome segments may be formalized as gene teams). The second step, that computes CARs and ancestral genome architectures, benefits from a combinatorial framework, centered around the Consecutive Ones Problem and an ubiquitous combinatorial structure called PQ-tree [25], well known and used in physical mapping [26],[27], and recently applied in other comparative genomics problems [28],[29]; in particular, in [22],[30],[31], PQ-trees were already considered to represent ancestral genomes. In our implementation of this second step, we follow the same principle as in [12]: we extract a maximum unambiguous subset of ancestral syntenies.

We apply our method on several datasets. We first consider the case of the ancestral boreoeutherian genome using a dataset obtained from the whole genome alignments available on the UCSC Genome Bioinformatics website [32]; from these alignments, we build sets of synteny blocks at different levels of resolution (we use from 322 to 1675 homologous markers). Our experiments show that the results of our method are quite constant, in the sense that they are very similar, independently of the chosen resolution. This reinforces the impression that algorithmic aspects may have an important impact on the differences in the results of [11],[12] discussed in [16]–[18], together with the differences of data acquisition and interdisciplinarity problems [18]. Moreover, the results we obtain are very close to the ones towards which cytogenetics methods tend to converge. As these are obtained from many more species and much expertise, we take it as a validation of the framework and method we propose. We performed intensive comparisons with other computational methods, and ran our method on several published datasets. Compared to the recently published method of Ma *et al.* [12], we obtain sets of CARs that are less well defined, as we propose a large set of possible ancestral boreoeutherian genome architectures, instead of only one, but better supported, as any proposed adjacency or segment is supported by at least one syntenic group that is conserved in at least two extant species whose evolutionary path in a phylogenetic tree contains the wished ancestral species. We also reconstruct an ancestral ferungulate genome architecture for the the same data as [11]. On this dataset, our method and the method of Ma *et al.* obtain similar results. The CARs are comparable to those of the ferungulate chromosomes from e-painting studies [33] that are ancestral boreoeutherian features, while the rearrangement-based method of [11] on the same dataset gives divergent results.

In the next section, we describe the general framework and how we implemented it to design a new method for ancestral genome reconstruction. We then describe the results of our method on the considered mammalian datasets. We use our reconstruction of possible genome architectures for the boreoeutherian ancestor at several levels of resolution to assess both the internal stability of our method and the consistency of its results when compared to other published ancestral genome architectures. We compare our results to the results proposed by cytogenetic methods and by the bioinformatics method of Ma *et al.* [12], that received some attention recently [18] as it was the first bioinformatics method that tended to agree well with cytogenetics. We conclude by a discussion on our results and methodology and describe several possible extensions of our framework.

## Results

### A General Methodological Framework and Implementation

We now describe more precisely the two steps of the framework, together with their implementation into an effective method for reconstructing a set of CARs. We separate the general principles from the implementation details to emphasize that there are many possible implementations: the method of Ma *et al.* [12] is one possibility, and we also propose a variant of our method targeted at analyzing datasets with less well defined outgroups.

#### Input: Species tree

The input of our method is a set of extant genomes, together with a phylogenetic tree *T* describing the evolutionary relationships between the species to which the genomes belong. The ancestral genome we want to construct is characterized by its position, as an internal node on the phylogenetic tree. Following [12], we assume that there is at least one outgroup species, that is, one species which is not a descendant of the ancestor whose genome we are reconstructing. This implies that the ancestral node has at least two branches towards its descendants (exactly two if the tree is fully resolved) and one branch towards the outgroup species. Additionally we may add branch lengths to indicate the relative *a priori* expected quantity of evolution. The method we describe relies on this phylogeny as we infer ancestral features only if they are supported by at least two species whose evolutionary path goes through the ancestral node (see paragraph “Detection of putative ancestral genome segments” below). While this principle is widely used by cytogeneticists to reconstruct ancestral karyotypes, no computational method so far has ensured this simple property.

**Figure 1:**
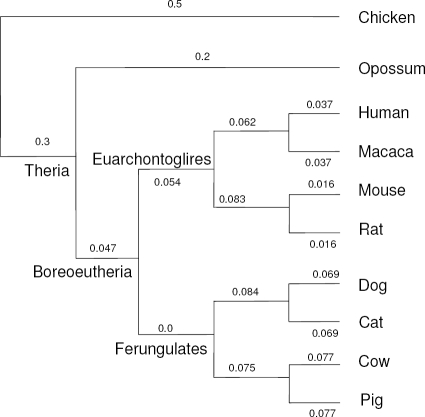
The phylogenetic relationships between studied species, taken from [34]. The branch weights are computed from the lower bounds on estimated times of species divergences from the same paper.

***Implementation.*** We consider three datasets, focusing on two ancestral nodes of the mammalian clade: the boreoeutherian and ferungulate ancestors. The choices were made according to the possibilities of comparisons of the obtained ancestors with former studies [5]–[7],[11],[12],[33]. The phylogenetic tree of all considered species is described in Figure 1, and the branch lengths were taken according to lower bounds from recent studies in paleontological dating [34].

#### Input: Representation of extant genomes

Following other approaches for ancestral genome reconstruction, we represent the genome of an extant species by a set of sequences of genomic markers. Each marker belongs to a family of homologous markers identified by a unique label. Such families of genomic markers can be defined in several ways: from annotated orthologous genes [10],[22],[35],[36], to whole genome alignments methods [37],[38] as in [10],[12], comparative maps [11] or virtual hybridization [39]. Each extant chromosome is an ordered sequence of markers, each marker being represented by the label of its family. If there are *n* family labels, we denote by 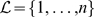 the set of all family labels (the markers alphabet).

***Implementation.*** We construct several datasets from the pairwise whole genome alignments between the human genome taken as reference, and the rhesus, mouse, rat, cow, dog, chicken, and opossum genomes, available on the UCSC Genome Bioinformatics website [32]. Pairwise synteny blocks between the human genome and the seven other extant genomes were computed from pairwise genome alignments, following the method described in [40],[41], for value of the parameters *max_gap* (the size of ignored micro-rearrangements or misplaced DNA segments) of 100 kb and of *min_len* (the minimum length of pairwise alignments with the reference genome) ranging from 100 kb to 500 kb (see details in Material and Methods). Then multispecies markers were computed using the human genome as a reference. For each value of the parameters *max_gap* and *min_len*, we kept the set of markers that are present in all eight genomes. In order to perform several comparisons with published methods, we also use datasets taken from Ma *et al.* [12], based on alignments at a 50 kb resolution and where markers can be duplicated, missing or overlapping in the outgroups, and from the supplementary material of Murphy *et al.* [11], based on human-mouse synteny blocks and comparative maps of seven mammalian genomes (human, mouse, rat, pig, cow, cat, dog).

#### Step 1: Detection of putative ancestral genome segments

The first step consists in detecting *ancestral syntenies*, that is, subsets of the alphabet of marker labels that are candidates to represent *contiguous markers in the ancestral genome*; this point is central in our framework (see Discussion) and is close to cytogenetic methods, though working with different data. The general problem of this first step reduces then to defining synteny conservation patterns along the species tree *T* that indicate a possible ancestral synteny, and to detecting such patterns.

***Implementation.*** We chose to follow a simple general principle: a group of genomic markers is possibly contiguous in the ancestor genome if it is contiguous in at least two extant species whose evolutionary path on the phylogenetic tree goes through the considered ancestral node. From then, several synteny conservation models between pairs of genomes can be considered to build ancestral syntenies: adjacent pairs of genes with the same orientation, as in [12],[36], or common intervals as in [22]. Here we use the following notions of conserved features: (1) gene teams with no gaps (also called maximal common intervals) [24], defined as maximal genome segments that have the same content in terms of genomic markers, (2) non-ambiguous unsigned adjacencies, and (3) approximate common intervals (used instead of maximal common intervals to analyze the dataset of [12]), defined as common intervals relaxing the condition of having exactly the same gene content (see Material and Methods for formal definitions).

As such ancestral syntenic groups can have very different conservation patterns in *T*, we associate to each of them a weight, based on the pattern of occurrence of this set of markers in *T* and on the branching pattern of *T*, following the weighting scheme used in [12] (see Material and Methods). This weight is a way to measure the extent of conservation of a given feature.

#### Step 2: Structuring ancestral features and PQ-trees

The output of the first phase is a set 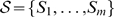 of *m* weighted, and pairwise different, ancestral syntenies. Each ancestral synteny is a subset of 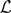 which contains genomic markers which are believed to be contiguous in the ancestral genome. The problem is then to group the markers of 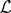 into CARs, and to order them inside these CARs, which, from a computational point of view, is very related to physical mapping problems [26],[27],[42]. (In physical mapping problems, markers representing the hybridization of probes are known but their relative order in the mapped genome is not known, and what is known, from hybridization with genome fragments, is that some sets of markers need to be contiguous; the problem is then to find an organization of the markers into chromosomes, such that all, or a maximum of subset of 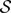 if it is not possible to handle all markers, are indeed contiguous in the resulting genome.) Intuitively, the conserved syntenic groups of *S*, that represent sets of possibly ancestral contiguous markers, can be seen as ancestral genome fragments that have evolved along *T* and are observed today conserved in at least two species. We then use an approach developed, first in the graph theory community (the Consecutive Ones problem was introduced by Fulkerson and Gross [43] to solve the problem of recognition of interval graphs, which, intriguingly, was motivated by another molecular biology problem in [43]!) and then applied to physical mapping problems, based on the *consecutive ones property* (C1P) and *PQ-trees*.

We encode *S* by an *m*×*n* 0/1 matrix 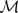 where row *i* represents *S_i_* as follows: 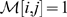 if marker *j* belongs to *S_i_* and 0 otherwise. Ordering markers into CARs consists in finding a permutation of the columns of the matrix 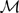, such that all 1's entries in each row are consecutive (also called a C1P ordering for 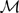). Finding such an order of the columns of 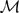 is not always possible, in particular if there are false positives in 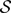, that is groups of markers that were not contiguous in the ancestral genome. Moreover, if there exists a C1P ordering of the columns of 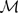, there are often several possible (sometimes an exponential number of) such orderings where all 1's are consecutive on each row. Every ordering represents an alternative possible ancestral genome architecture.

In the case where there exists a C1P ordering for 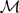, all C1P orderings can be represented in a compact way, using the *PQ-tree* of 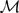, denoted 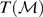. We now provide a short description of the important properties of this structure with respect to C1P orderings (a complete formal description is given in Material and Methods). 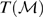 is a tree with three kinds of nodes: leaves, P-nodes and Q-nodes. The leaves are labeled by 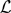, in such a way that each 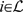 labels exactly one leaf of 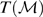. P-nodes and Q-nodes are internal nodes, both with a total order on their children. The main property of 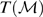 is that any C1P ordering of 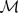 can be obtained from 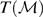 by reading, from left to right, the leaves labels of 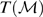 after choosing for each node *N*, independently of the other nodes, (1) an arbitrary order for the children of *N* if *N* is a P-node, or (2) to reverse or not the order of the children of *N* if *N* is a Q-node.

**Figure 2:**
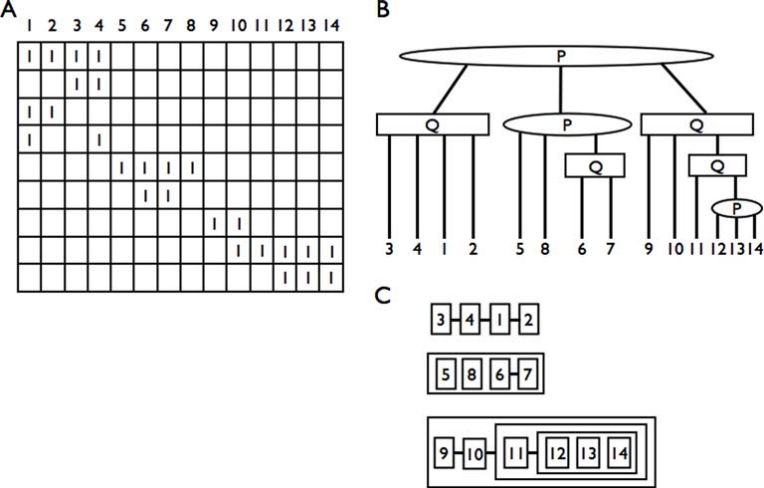
Representation of a family of sets with the consecutive ones property. (A) A matrix 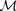 with the consecutive ones property. (B) The corresponding PQ-tree 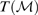, where P-nodes are rounded and Q-nodes are square. 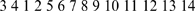 and 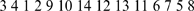 are two possible C1P orderings for 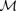, among 13824 possible C1P orderings. 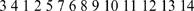 is not a C1P ordering for 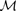: columns 6 and 7 need to be consecutive as they are consecutive children of a same Q-node. (C) An equivalent representation of 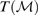 which highlights all ancestral genome architectures that correspond to C1P orderings for 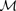: each row corresponds to a chromosomal segment represented by a child of the root, two glued blocks have to be adjacent in any ancestral genome architecture and sets blocks that float in the same box have to be consecutive in any genome architecture but their order is not constrained. Here we see three ancestral chromosomal segments: the first one, which contains markers 1 to 4 is totally ordered; the second one contains markers 5 to 8, with only constraint that markers 6 and 7 are adjacent; the third one contains markers 9 to 14, with 9 and 10 being adjacent, 11 being adjacent to a block that contains 12, 13 and 14 with no order between these three markers. Hence, 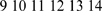 is a possible order for this last segment, but not 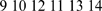 as 11 is inserted inside the block that contains 12, 13 and 14. All 13824 possible C1P orderings (possible ancestral orderings) are visible on this representation.

An important property of the framework we describe is that, if all markers are true orthologs and if all 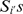 are true positive, that is, were indeed contiguous in the ancestral genome, then there exists a C1P ordering of the markers of 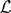. In that case, 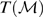 encodes in a compact way all possible C1P orderings of the columns of 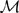 and then all alternative genome architectures we can deduce from 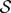: the root of 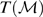 is a P-node, children of the root represent CARs, where Q-nodes describe fixed orderings, up to a reversal, while P-nodes except the root describe subsets of markers that have to be contiguous but where there is no information to fix a relative order (see Figure 2 for an illustration). A linear representation of the PQ-tree allows to present the set of whole C1P solutions in a chromosome-like form (Figure 2c). In a PQ-tree, two markers define an *adjacency* if they are consecutive siblings of a Q-node.

**Figure 3:**
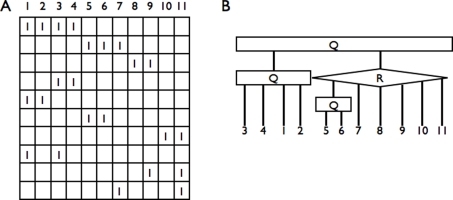
Representation of a family of sets without the consecutive ones property. (A) A matrix 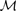 without the consecutive ones property. (B) The corresponding generalized PQ-tree, where there is a single R-node represented by a diamond shape labeled R. The only R-node is due to the rows 1, 2, 6, 7 and 9 of 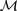 that define a sub-matrix that is not C1P, while the submatrix defined by the remaining rows is C1P.

Finally, if 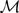 is not C1P, we can still represent some partial information from it using a structure called the *PQR-tree* in [44] or *generalized PQ-tree* in [45], that we also denote by 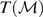. It contains a fourth kind of nodes, called *degenerate nodes* or *R-nodes* which represent disjoint subsets of 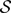 that are not C1P. Hence, 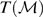 extracts parts of 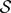 that are unambiguous and can be used directly to define CARs (the P-nodes and Q-nodes of the generalized PQ-tree), unlike the ambiguous parts of 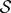 that contain non-ancestral features (the R-nodes). An illustration of such a case is presented in Figure 3. It is then a first level of representation of CARs, that contains possible ambiguous information and generalizes the successor and predecessor graphs of [12]. Computing 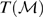 can be done efficiently (see Material and Methods).

#### Step 3: Clearing ambiguities and constructing CARs

As pointed above, if 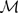 is C1P, there is no indication that some features of 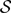 are not ancestral, so we directly output the possible ancestral genomes as the PQ-tree 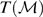. However, if 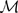 is not C1P, then we know that some sets of markers in 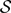 are false positive and were not contiguous in the ancestral genome. There can be several reasons: errors in constructing homologous markers (errors in the assemblies, paralogies inferred instead of orthologies), incomplete syntenies resulting from convergent loss of markers, convergent fusions of chromosomal segments in several lineages for example. As in physical mapping [46], depending of the kinds of errors that have to be removed, there are several ways to remove ambiguous information present in 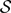 such as discarding some markers or features, or splitting possibly chimeric sets of markers in two or more subsets. After ambiguous information has been removed, there remains a subset 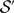 of 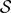 that defines a C1P matrix 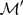 and a PQ-tree 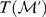 that represent all possible genome architectures compatible with 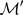.

***Implementation.*** In our implementation we did not consider the option of discarding markers, that is, removing columns of the matrix. Indeed, as we considered DNA alignments at a resolution of at least 100 kb, taking care about possible paralogies by eliminating segmental duplications and repeated elements, we have a good confidence in the set of orthologous markers. We then clear ambiguities by removing elements from 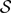, i.e. rows from 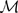 that represent possibly non-ancestral syntenies. More precisely, we rely on the following combinatorial optimization problem: find a subset of 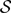 of maximum cumulative weight, such that the matrix of this subset is C1P. This problem, which generalizes the approach used in [12], is NP-hard (it generalizes the traveling salesman problem). We solve it using a branch-and-bound algorithm based on a greedy heuristic inspired from [12] (see Material and Methods). We take the proportion of rows that have to be deleted as an indicator of the level of *ambiguity* of a dataset.

### Reconstructing Ancestral Mammalian Genome Architectures

In this section, we first report the results of our method in reconstructing the architecture of the boreoeutherian ancestral genome from five datasets, at different levels of resolution, that we computed from whole genome alignments. Next we report results based on the original dataset used in [12] and on the ferungulate ancestral genome from the dataset of [11]. All data and results discussed in this section are available on a companion website: http://lbbe-dmz.univ-lyon1.fr/tannier/ploscb2008_supmat/.

### The Boreoeutherian Ancestor with a Dataset Constructed from UCSC Genome Browser Whole Genome Alignments

We computed five datasets, with parameters *max_gap* = 100 kb and *min_len* = 100 kb (1675 markers), 200 kb (824 markers), 300 kb (510 markers), 400 kb (406 markers) and 500 kb (322 markers). Their coverage of the human genome goes from 2173 Mb (*min_len* = 100 kb) down to 1487 Mb (*min_len* = 500 kb).

#### Computational characteristics of the CARs inference method

From a computational point of view, these five datasets seem to contain very little ambiguity. For example, with *max_gap* = 100 kb and, *min_len* = 200 kb, only 14 of the 1431 ancestral syntenies detected during the first step needed to be discarded to clear all ambiguities in the 0/1 matrix. The branch-and-bound algorithm finds a provably optimal solution in a very small amount of time. With other values of *min_len*, the computational characteristics were similar (very few ancestral syntenies need to be discarded to clear ambiguities). This is important to remark, as it lowers the influence of the optimization step in the framework. This step is the most subject to arbitrary choices, so we think the less it relies on optimization, the more the method is reliable.

#### Properties of the different ancestral genome architecture proposals

In Table 1, we see that generally the number of CARs obtained decreases as *min_len* increases, which is expected as larger synteny blocks hide more rearrangements and misassemblies that could prevent ancestral syntenies to be detected. The number of CARs tends to converge towards the accepted number of 23 chromosomes in the boreoeutherian ancestral genome, despite the presence of 29 CARs at resolution 300 kb due to three markers that do not belong to any ancestral synteny and define each a CAR. The correspondence between the CARs and human chromosomes is very stable: aside of chromosome 1, for whom it seems to be hard to infer the ancestral structure (it spans one CAR at a resolution of 100 kb, 2 CARs at 200 kb, 4 CARs, including one reduced to a single marker at 300 kb, 3 CARs at 400 kb and 2 CARs at 500 kb; note however that the history of human chromosome has not been easy to write by cytogeneticists. While it is in two pieces in some works [7], the study of Murphy *et al.* [47] states its probable unichromosomal history in placental mammals.), for all other human chromosomes, the number of spanned CARs is stable or decreases as the resolution decreases from 100 kb to 200 kb. It is due to the fact that at lower resolution, due to the lower coverage of genomes, some large part of human chromosomes do not contain any marker and do not map to any CAR. The only exceptions are due to two markers of chromosomes 8 and 19, at resolution 300 kb that define each a CAR, because the markers that were syntenic with them in other species at resolution 200 kb are not conserved at 300 kb; note also that these two markers both disappear when *min_len* = 400 kb, which explains that we find again 26 CARs.

**Table 1:** Characteristics of the datasets based on the UCSC alignments, and the obtained reconstructed boreoeutherian ancestral genome architectures with our method.

*min_len* (kb)	Markers	CARs	Human cov. (Mb)	Adjacencies	Chromosomal syntenies
100	1675	29	2667	1604	1-4, 3-21, 4-8, 7-16, 12-22, 12-22
200	824	26	2511	778	3-21, 4-8, 7-16, 12-22, 12-22, 14-15, 16-19
300	510	29	2179	449	3-21, 4-8, 7-16, 12-22, 12-22, 14-15, 16-19
400	406	26	2186	372	3-21, 4-8, 7-16, 12-22, 12-22, 14-15, 16-19
500	322	24	1796	260	3-21, 5-8, 7-16, 12-22, 12-22, 14-15, 16-19

The “Markers” column describes the number of synteny blocks of each dataset. The “Adjacencies” columns describes the number of adjacencies in the ancestral genome architecture. In the right-most column, a set of numbers linked by - indicate a CAR that contains markers that belong to the corresponding human genomes. The “human coverage” is the portion of the human genome that is covered by sets of markers that are consecutive on a CAR and on the human genome, although possibly in different orders, expressed in Mb.

As *min_len* increases, the coverage of the extant genomes decreases, and beyond the value of *min_len* = 500 kb, the missing parts become more and more visible, so the reconstruction becomes less reliable, as the ancestor covers only a small part of the extant genomes. The coverage of the human genome by the CARs, containing several contiguous sets of markers goes from 2667 Mb (for *min_len* = 100) to 1796 Mb (for *min_len* = 500) and is larger than the coverage by the markers only.

For chromosomal syntenic associations in the inferred ancestral genome architecture between some human chromosomes, we can also see that the results we obtain are very consistent, and in general do not propose CARs which disagree with previous cytogenetics studies [14],[16]. The only differences within the chromosomal associations are the synteny between human chromosomes 1 and 4, seen with *min_len* = 100 kb only, a synteny between human chromosomes 5 and 8, observed only with *min_len* = 500 kb, and an association between human chromosomes 4 and 8 that is not present with *min_len* = 500 kb. This last fact is linked to the resolution as the only marker of human chromosome 8 that participates to the association chr4-chr8 at *min_len* = 400 kb disappears at *min_len* = 500 kb; we discuss the two other associations in relation to the notion of support below. Other differences between the results obtained with the different values of *min_len* mostly involve the number of CARs corresponding to human chromosomes 1 and 2.

**Figure 4:**
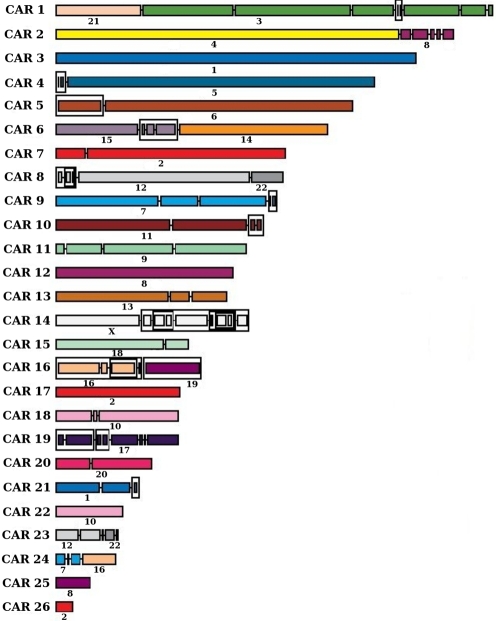
The ancestral genome architecture obtained with the dataset constructed from the UCSC whole genome alignments, with parameters *max_gap* = 100 kb and *min_len* = 200 kb. Segments of a given color represent sequences of genomic markers that are colinear in the inferred CARs and in a human chromosome called *conserved segments* (corresponding human chromosomes numbers are indicated with each conserved segment). The size of conserved segments in the figure is proportional to the sum of the size of the the human genome that is covered by the synteny blocks they contain. The nodes of the PQ-tree are represented: children of a linear (Q) node are linked by a small segment, while children of a prime (P) node are grouped together with a rectangular frame.

With values of *max_gap* = 100 kb and *min_len* = 200 kb, a higher resolution than the one used in [10],[11], we obtain 26 CARs presented in Figure 4. We can compare the obtained CARs with the previously published boreoeutherian ancestors, in the light of some recent discussions on these results [16]. We recover ancestral segments that are very close to cytogenetic studies: all the 26 segments of the *max_gap* = 100, *min_len* = 200 kb dataset are indeed segments with which all cytogenetic publications agree [2]–[7], and this is the first reported bioinformatics study which verifies this. We just miss two or three adjacencies according to the studies: some are probably due to the incompleteness of our data in terms of covering of extant gnomes by universal synteny blocks (human chromosome 2 is cut into three pieces and human chromosome 1 is cut into two pieces in our reconstruction, whereas it was probably a unique piece in the ancestor [47]), and others are debated in the community (adjacency between human chromosome arm 10p and an ancestral chromosome 12–22 [5],[6], or between chromosome 1 and a segment from chromosome 19 [6]). We obtain similar results for values of *min_len* = 300 kb and *min_len* = 400 kb, with minor differences about the coverage of human chromosomes 1 and 2 for *min_len* = 400 kb, and the presence of three small CARs containing each a single marker with *min_len* = 300 kb.

#### Adjacencies in ancestral genome proposals: support and stability

We report in Table 1 the number of adjacencies; this number indicates how well defined the ancestral genome architectures are, as the markers that are not in an adjacency belong to sets of markers that are children of a P-node and whose relative order is not known. There are relatively few such markers (between 6% and 20%), which means that the ancestral genome architectures are quite well defined.

We now define the support of an adjacency between two markers in an ancestral genome as the number of ancestral syntenies which contain these two markers. The adjacencies of our five ancestral genomes are in general well supported as in total we find 14 adjacencies that are supported by a single ancestral synteny (2 for *min_len* = 100 kb, 4 for *min_len* = 200 kb, 3 for *min_len* = 300 kb, 3 for *min_len* = 400 kb and 2 for *min_len* = 500 kb). Among these minimally supported adjacencies, only two of them imply a chromosomal association: between human chromosomes 4 and 8 with *min_len* = 400 kb and between human chromosomes 5 and 8 with *min_len* = 500 kb (this last one is supported by a single gene team common to the rat and opossum genomes and it involves a single marker of length 1600 kb in human chromosome 5), which raises some doubts on the validity of this chromosomal association that is found only at the highest value of *min_len* and not well supported. On the other hand the association between human chromosomes 1 and 4 with *min_len* = 100 kb is supported by 7 gene teams, but involves a single marker of human chromosome 4.

One of the reasons for computing several datasets at different resolution levels but based on the same set of initial alignments was to assess the stability of our method. To do so, given an ancestral adjacency obtained at a given level of resolution (say *min_len* = 100 kb) we say that it is *not conserved* at a lower resolution (say *min_len* = 200 kb) if the markers defining this adjacency are neither in the same marker or in adjacent markers at this lower resolution, and we say that it is *weakly conserved* if the markers stay on the same CAR, but not adjacent, at the higher resolution (see Material and Methods for precise definitions). We compared all 10 pairs of ancestral genome architectures for all pairs of values of *min_len*, and we found only 2 non-conserved adjacencies (with no surprise they belong to the ancestor computed for *min_len* = 100 kb) and 141 weakly conserved adjacencies. For these last ones, the markers are in general close in the CAR which contains them (on the average the gap between such pairs of markers on a CAR contains approximately 3 blocks, with only 32 such pairs being separated by more than 5 blocks).

#### Considering common intervals in defining ancestral syntenies

To assess the impact of considering common intervals to define ancestral syntenies instead of adjacencies, we applied our method on the same datasets but using only conserved adjacencies (i.e. pairs of markers adjacent in two genomes whose evolutionary path goes through the ancestral node), without accounting for the orientation of the markers. We obtain, for every value of *min_len*, a larger number of CARs (between 36 and 42 CARs), with many CARs containing few markers. However, the computational characteristics and the stability of the proposed sets of CARs are similar: the datasets contain little ambiguity and the computed adjacencies and human chromosomal associations are very stable, these last ones agreeing with the ones we described above. This points the importance of using larger syntenic sets to infer more precise sets of CARs.

#### Comparison with the method of Ma *et al*

The main differences between our method and the one of Ma *et al.* are the restriction on ancestral features to well supported ones and the addition of common intervals in the ancestral features, which implies that the combinatorial framework switches from Path Partitioning problems in graphs to the more general Consecutive Ones problem. So we add more information, and ask it to be more reliable. To assess if this theoretical consideration has some effect, we ran the method described in [12] on the datasets we have constructed, using the software available at http://www.bx.psu.edu/miller_lab/car/, in the frozen version used in the paper [12]. We report the results in Table 2. For every proposed adjacency between two markers *i* and *j*, we say that it is *weakly supported* if there is no ancestral synteny in 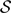 (the set of ancestral syntenies computed by the first step of our method) which contains both *i* and *j* (by construction, every adjacency computed with our method is supported by at least one ancestral synteny). We also say that an adjacency is *common*, if it is also present in the CARs obtained with our method. Table 2 shows that most of the differences between the two methods are due to adjacencies that are obtained with the method described in [12] but are not supported by an ancestral synteny as we define them. We also notice that a small number of differences (which represent a very small percentage of all adjacencies) may have some important implications in terms of inferred chromosomal syntenic associations between human chromosomes in the ancestral genome. Moreover, the two methods differ slightly in terms of stability: comparing the ancestral genomes obtained with all five values of *min_len*, we found that 12 adjacencies were not conserved (including those that induced the chromosomal syntenies that are not constant at all resolution levels) and 88 adjacencies were weakly conserved. This is expected as our new methods provides more support and stability, but less well defined CARs due to the simultaneous presentation of a large set of solutions.

**Table 2:** Characteristics of the reconstructed genome architectures of boreoeutherian ancestral genomes with the method of Ma *et al.* [12] and our synteny blocks.

min_len (kb)	markers	CARs	Weak adj.	Common adj.	Human chromosomal syntenies
100	1675	31	6	1596	1-16, 3-21, 4-8, 12-22, 12-22, 12-22
200	824	34	5	759	1-10, 3-21, 4-8, 12-22, 12-22
300	510	37	7	437	1-10, 1-17, 3-12-21, 4-8, 12-22, 12-22
400	406	36	7	353	2-4, 2-22, 3-12-21, 12-22
500	322	37	6	249	2-4, 3-12, 3-21, 12-22

The weak adjacencies are the inferred adjacencies that are not supported by at least two species whose evolutionary path contains the boreoeutherian ancestor. If this criterion is to be followed, this number is to be added to the number of CARs.

### The Boreoeutherian Ancestor from Ma *et al.*'s [12] Data

We also analyzed the dataset of 1338 conserved segments used in [12], downloaded from the website http://www.bx.psu.edu/miller_lab/car/. It has the impressive property that these conserved segments span slightly more than 94% of the human genome based on alignments at a 50 kb resolution level. On the other hand, it considers less species, an unbalanced phylogeny (one of the branch from the ancestral node contains a single species, the dog, while the other branch contains three species, human, mouse and rat) and the segments are less well defined in the outgroups: they can be duplicated (due to ambiguous orthology signal), missing or overlapping. In order to analyze this challenging dataset, we modified our method, to handle the different combinatorial nature of segments in outgroups, and we chose to define ancestral syntenies in terms of conserved adjacencies and approximate common intervals which do not require the exact same markers content and allow for duplicated markers (see Material and Methods). This illustrates the generality of our framework: the way to define ancestral syntenies and the type of dataset is flexible. While we prefer to present the results with our own dataset due to its better proximity to the C1P property, we performed our method on this dataset for the method comparison to be as exhaustive as possible.

The set of possible ancestral syntenies contains 2515 subsets of segments, and 208 needed to be discarded in order to clear all ambiguities and get the C1P property. This shows that by relaxing the definition of ancestral synteny by allowing inexact content, we introduced a large number (at least 10%) of false positives (i.e. groups of segments which were not consecutive in the ancestral genome). We obtained an ancestral genome with 35 CARs, 1281 adjacencies and the following human chromosomal associations: 3-21, 4-8, 12-22, 12-22, 14-15, to compare to 29 CARs and 1309 adjacencies and the same human chromosomal associations in [12]. Among our 1281 adjacencies, 1077 are present in the 1309 adjacencies obtained with the method of Ma *et al.*. As before, we define a weak adjacency as an adjacency obtained by the method of Ma *et al.* whose segments are not included in any of our ancestral syntenies: 8 of the 1309 adjacencies obtained in [12] are weak. Among these adjacencies are several human or rodent or dog specific adjacencies. The fact that we have significantly fewer common adjacencies while the adjacencies of Ma *et al.* are still well supported can be explained by the fact that some adjacencies inferred in [12] are supported by false positive ancestral syntenies, which are much more frequent with this dataset than when using or own datasets of universal markers, where we used several filters to eliminate them. For example, by assessing the support of the adjacencies in the 29 CARs obtained by Ma *et al.* in terms of the ancestral syntenies conserved after our second phase, which produces a C1P matrix, 21 are not supported, and the general level of support of adjacencies decreases in general.

### The Ferungulate Ancestor from Murphy *et al.* Synteny Blocks

We also tested our framework on the ferungulate ancestor based on the dataset of Murphy *et al.* [11]. This dataset contains seven genomes, which are represented by 307 synteny blocks that cover 1343 Mb of the human genome [11]. It is hazardous to reconstruct boreoeutherian ancestors with this dataset, because there is no outgroup for the boreoeutherian clade here, but it is interesting to use this dataset to compare several methods on a dataset we did not construct. We ran both our method and the one of Ma *et al*. [12] on this dataset and compared the inferred genome architectures. We include in the comparison the results obtained by Murphy *et al.* [11] on the same dataset, and those of Kemkemer *et al.* [33] obtained independently by a computational method called e-painting, see Table 3. The ancestral genome architecture we propose is based on 457 ancestral syntenies from an initial number of 461, and here again the dataset seems to contain very little ambiguity.

**Table 3:** Characteristics of four inferred ferungulate genomic architectures. The first three use a set of markers taken in the supplementary material of Murphy *et al.* [11].

Method	CARs	Adjacencies	Human chromosomal syntenies
New method	24	250	1-10, 3-21, 4-8, 7-16, 12-22, 12-22, 14-15, 16-19
Ma *et al.* [12]	38	269	2-7-16, 3-21, 4-8, 12-22, 14-15, 16-19
Murphy *et al.* [11]	24	283	1-10, 1-22, 2-20, 3-21, 4-8, 5-19, 7-16, 12-22, 14-15, 16-19
Kemkemer *et al.* [33]	23	-	1-3-19-21, 4-8, 7-16, 12-22, 14-15, 16-19

It consists in 307 markers covering 1343 Mb of the human genome. We have run our program and the one of Ma *et al.* [12], while taking the published results of Murphy *et al.*. The last method is with a different set of markers, constructed by e-painting methods. We copy the chromosomal syntenies published in [33].

Some syntenies obtained belong to the boreoeutherian ancestor, and others are ferungulate specific. The synteny between human chromosomes 5 and 19 is inferred only by Murphy *et al.* (where it is not marked as weak, which means that it was found in all alternative genome architectures) but not by our method. However, it is due to an adjacency between two synteny blocks that is not found in any of the ancestral syntenies we detected in the first step of our method, and is found only in the pig genome. The synteny between human chromosomes 1 and 22 is inferred only by Murphy *et al.*, where it is marked as weak. It is due to an adjacency that is not found in any genome, nor supported by any of our ancestral syntenies. The same holds for the synteny between human chromosomes 2 and 20 (which is not weak according to Murphy *et al.*), and seems to be more rodent-specific. The synteny between human chromosomes 1 and 10 was inferred by MGR and our method, and considered weak by Murphy *et al.*, and is supported by three of our ancestral syntenies that have significant weights. The synteny between human chromosomes 2 and 7, which is found only by the method of Ma *et al.* is due to an adjacency that is found only in the pig and is not supported by any of our ancestral syntenies. We can also note that among the 250 adjacencies inferred by our method, only 196 are common with the results obtained with the methods of Ma *et al.* and Murphy *et al.*, while 240 are common with the ancestor obtained with the method of Ma *et al.* and 204 are common with the ancestor proposed by Murphy *et al*. We have only the boreoeutherian syntenies in common with Kemkemer *et al.* [33], and those that are supposed to be ferungulate specific all disagree (we don't recover the giant chromosome 1-19-3-21, and recover 1-10 instead).

## Discussion

We proposed a general model-free framework for reconstructing ancestral genome architectures from current genomic marker orders. We implemented this framework in a method that considers adjacencies and common intervals in extant genomes and applied our method on two ancestral genome reconstruction problems: the boreoeutherian ancestor, from a set of homologous markers we computed from UCSC whole genome alignments [32] and a dataset proposed in [12], and the ferungulate ancestor from the synteny blocks defined in [11]. We believe that our experimental results mark a progress as compared to previous bioinformatics studies, and that the framework we propose is a useful tool to compare methods.

### Convergences and Divergences of the Ancestral Genome Reconstruction Methods

We perform here a comparative analysis of different methods for the reconstruction of ancestral genomes, independently of the type of data used for these reconstructions. For the boreoeutherian ancestor, Ma *et al.* [12], with their own set of markers called conserved segments, recovered 29 CARs, with 8 “weak adjacencies”. Those adjacencies correspond to features that are only present in human and mouse for example, which would more account for an euarchontoglire feature, or even only in human (as the junction of both parts of human chromosomes 10 or 16 for example). In contrast, at a resolution of 200 kb and with universal synteny blocks, we infer 26 CARs, which is comparable, but no such weakly supported adjacency is inferred. At the resolution of 50 kb, with Ma *et al.* data, we infer 35 CARs, which compares to 29 CARs plus 8 weak adjacencies. Moreover, all our chromosomal syntenies, at several resolution levels, are also supported by cytogenetic studies, but the fusion of a synteny block of human chromosome 4 with a segment of human chromosome 1 that is found only at high resolution (*min_len* = 100 kb). The method of Ma *et al.* gives 31 to 37 CARs on our datasets, with a significant number of weak adjacencies, as well as some variations in terms of human chromosomal associations. The most likely explanation for the difference between the two methods lies in methodological reasons, primarily the way ancestral syntenies are defined (adjacencies computed through a Fitch-like approach in [12], see below for a discussion on that topic), rather than to the dataset itself as the way we compute synteny blocks are very similar, even if we conserve only blocks that are present in all genomes. Nevertheless, the results obtained both by our method and Ma *et al.* method, which both rely on model-free algorithmic principles, like cytogenetics methods but on other kind of data, strongly agree with cytogenetics results.

We also tested our method on the ferungulate ancestor and compared our results with the ancestor inferred through a rearrangement-based method in Murphy *et al.* [11]. With the method Murphy *et al.*, based on a genome rearrangement model and MGR [8], the results diverged from the cytogenetics data and provoked the discussion in [16]–[18]. Using the same synteny blocks as Murphy *et al.*, we found 24 CARs, all of which are chromosomes of the boreoeutherian ancestor, except a fusion of the homologs of human chromosomes 1 and 10, which seem to be ferungulate-specific, and was also inferred by MGR. None of the other chromosomal syntenies proposed by [11] were recovered by our method, or the Ma *et al.* method. However, the number of common inferred ancestral adjacencies points out that our method and the method of Ma *et al.* compute similar ancestral genome architectures, which are different from the one proposed by MGR, despite the fact that this last one has 24 CARs, as with our method. We believe that this three-way comparison indicates that the differences discussed in [16],[17] are partly due to the methods themselves, and more precisely to the fact that MGR is a rearrangement-based method, whereas all the others are model-free.

### Methodological Comments

We now summarize the main methodological features of the framework we propose, and discuss them, as well as some possible extensions. We propose to decompose the process of ancestral genome architecture inference into three steps: detection and weighting of ancestral syntenies, representation as a 0/1 matrix and a generalized PQ-tree, clearing ambiguities and representation of a set of alternative genome architectures as a PQ-tree. Although these three steps are performed independently, the implementation choices for each of them can have important consequences on the other ones, as we discuss below. We implemented this method using (1) unique and universal synteny blocks, which appear once in each genome, (2) ancestral syntenies defined as unambiguous adjacencies and maximal common intervals (or gene teams) which are present in at least two genomes whose evolutionary path along their phylogeny meets the considered ancestral species and (3) a combinatorial optimization approach, based on the Consecutive Ones Submatrix Problem, to clear ambiguities. The comparison of our method and the one of Ma *et al.* [12] through the prism of this framework highlights the important effects of some methodological choices on ancestral genome proposals. We discuss below these choices on the combinatorial nature of the considered sets of genomic markers, the definition and computation of ancestral syntenies, and the method to clear ambiguities.

#### The model-free approach

By following the model-free approach, we come close to the results of cytogenetics studies. Of course, it might not be a surprising finding, since we claimed at the beginning that we were trying to implement some of the principles that were used in the cytogenetics studies. But this is still a result, since it has never been reported before that with different types of data and a much reduced species sample, the same principles would lead to the same results.

The quality of the sequences and their assemblies, and the heuristics used to align them might have caused a divergence between the results in spite of the similarity of the reconstruction principles. We see here that this divergence is limited, and this can be explained by the model-free approach. Indeed, first, we do not try to force an explanation for a misassembly through an evolution scenario (see [48] for such an example). In addition, we consider ancestral features in terms of common intervals that are shared by at least two species whose evolutionary path goes through the ancestral node. So in order for such a putative ancestral synteny to be a false positive it would require that it is present in another species, which is not likely for a misassembled contig, provided the assemblies were done independently.

As an illustration, we tested our method on two different assemblies of the cow genome, bosTau3, which was the only one available when we started this study, and bosTau4, which is the one presented for the final results. These assemblies are quite different: as an example, we found 2796 homologous markers with parameters *max_gap* = 100 kb and *min_len* = 200 kb with bosTau3, and only 824 such markers with bosTau4, which witnesses a substantial progress in the cow genome assembly. But the boreoeutherian CARs are very similar with both versions. This indicates that the model-free method with common intervals is up to some point resistant to misassemblies. It can be well explained by the common interval model: contrarily to adjacencies, this is independent from the order of the markers in an ancestral group. Whether the cow genome presents markers ordered A B C D or A C B D due to a misassembly, the ancestral synteny in terms of content {*A*,*B*,*C*,*D*} will be captured by a common interval. The differences between the assemblies have however an impact on the possible resolution of an ancestral genome: while we obtain 26 CARs with parameters *max_gap* = 100 kb and *min_len* = 200 kb with the bosTau4 assembly (these are the CARs of Figure 4), we only obtain as good results from *min_len* = 400 kb with the bosTau3 assembly, thus with lower resolution and coverage.

#### Detecting ancestral syntenies

We emphasize that, in our opinion, the first step, which aims at computing a set of syntenic groups that are possibly ancestral, is essentially a feature detection phase and does not require to rely on combinatorial optimization. Current existing methods rely on methods inspired from the Fitch-Hartigan algorithm, as in [12],[22],[30]. These methods implicitly try to minimize the number of gains and losses of features along the species tree *T*, following then a parsimony model of evolution that can be very sensitive to the branching pattern of *T*. For example, in [12], due to the chosen taxonomic sampling and Fitch-based approach to define putative ancestral syntenies, all dog adjacencies will be considered as possible ancestral adjacencies. Weighting characters is a possibly more flexible approach to assess their conservation.

#### Handling duplicated and non universal markers

In order to analyze the original dataset of [12], we also show how our framework can be implemented to still define ancestral syntenies in terms of common intervals while accommodating less well defined synteny blocks in outgroup genomes, due to duplicated, missing or overlapping synteny blocks. To this aim, we use approximate common intervals. Note moreover that there are several algorithms to compute efficiently conserved syntenic groups between pairs of genomes with duplicated markers (see a survey in [49] for example), or duplicated segments followed by intensive losses in both copies (see [50]), which could be used instead of the algorithm to detect approximate common intervals we used.

However, what is compulsory in the framework we propose is that the ancestral genome contains exactly one marker of each marker family; indeed, otherwise we cannot use tools such as the notion of consecutive ones property of 0/1 matrices and PQ-trees, which are central in our framework. From that point of view, it would be interesting to extend our approach to problems of inferring a pre-duplication ancestral genome architecture, which has been considered in some rearrangement-based recent works [51]–[53]. Solutions in physical mapping techniques are also mentioned in [54]. Another approach, which has been followed recently when using gene families instead of synteny blocks would be to consider the gene trees of the gene families and the gene tree/species tree reconciliation to infer the ancestral gene content and orthology relationships [54].

#### Definition of ancestral syntenies, 0/1 matrices and PQ-trees

The link between the combinatorics of PQ-trees and 0/1 matrices is the main limitation of our approach, as it only captures certain types of ancestral syntenic features, and prevents to infer differentiated duplicated markers in the ancestral genome. For example, some common features of extant species are not captured by common intervals (gene team [23] with gaps). We would probably detect a significant amount of approximate ancestral syntenies by considering some amount of gaps in the detection phase [49],[55]. But the combinatorial nature of the reconstruction phase radically changes in this case, as naturally we would like then to consider possible gaps in the rows of the 0/1 matrix that represents ancestral syntenies after reordering the columns of this matrix. This is illustrated by the amount of ancestral syntenies that need to be discarded when they are defined in terms of approximate common intervals for example, which differs significantly from using exact common intervals.

When considering only 0/1 matrices, related problems have been considered as in [56], but they are not related any more to PQ-trees, which are important as they represent a set of alternative ancestral genome architectures, an important property of the framework we propose. The decision problem of “consecutive ones with allowed gaps” is still open. In this problem, each line of the matrix has to have consecutive ones, except that between each pair of ones, a fixed number of zeros is allowed. It is the extension of the C1P problem which is closest to the gene teams formalism. It relates to bandwidth in graphs [57], where it has a polynomial solution for maximum gaps of 2 (and more generally, if the maximum number of allowed gaps if fixed), but no generalization to matrices is known. There is then still an important theoretical work to do on the combinatorics of PQ-trees and of their extension to non-contiguous ancestral syntenies, which would be important to implement the framework we propose in order to handle more ancient and more rearranged genomes.

#### Clearing ambiguities in ancestral syntenies

In the method we propose, we decided to clear ambiguities in the set of detected ancestral syntenies by discarding the minimum amount (in terms of weight) of such syntenies in order to have a C1P matrix and then a PQ-tree. In fact we then made two choices: removing the minimum amount of information, and considering that only rows of the matrix may be discarded.

The bias induced by choosing to apply a combinatorial optimization approach is that we are likely to conserve, in the resulting matrix, false positive ancestral syntenies (for example if there are two false positive ancestral syntenies that have the same weight, and the presence of both contradicts the consecutive ones property, but not the presence of either of the two). Another approach was described in [30], where the notion of a *conflicting set* of syntenies was defined as a set of syntenies that is ambiguous but such that discarding any of them leaves a non-ambiguous set of syntenies. It was then proposed to discard all syntenies of such a group. This is what we do with adjacencies in the first step of our method, mostly because such conflicting sets are easy to detect with adjacencies, unlike with common intervals, and because we expect that true ancestral adjacencies should also be supported by larger syntenies that will be detected as maximum common intervals. With our data, such an approach would have been very extreme, as preliminary studies of ancestral syntenies that belong to the R-nodes of the generalized PQ-tree showed that almost half of such ancestral syntenies belonged to at least one conflicting set (data not shown). However, using a sampling method, it seems that only very few of these syntenies belong to many conflicting sets. It would then be interesting to apply a cut-off approach where all ancestral syntenies that belong to a large proportion of the conflicting sets present in a given R-node are discarded. However, to implement such an approach, the combinatorics of conflicting sets with general 0/1 matrices needs to be better understood (work in progress).

The second choice we made is the optimization criterion. There are several ways to handle conflicts in a 0/1 matrix that is not C1P (see [58] for example): removing rows (i.e. ancestral syntenies), columns (genomic markers), splitting rows (to account for possible chimeric ancestral syntenies) or even reverting some cells from 0 to 1 or 1 to 0 (to account for approximate syntenies). It is important to notice that choosing one of these approaches should be related to the nature of the errors expected to be found in the set of ancestral syntenies (see [46] for an example of this principle in the case of physical mapping). Based on our definition of genomics markers as synteny blocks computed from whole genome alignments using quite stringent criteria, we considered that orthology relations were correct (even if we found one possible false positive with *min_len* = 100 kb), which did not justify to remove columns. Similarly using maximum common intervals, that is genome segments with the same content, prevents from expecting to have to deal with reverting cells of the matrix. Finally, in the case of chimeric ancestral syntenies (i.e. groups of two or more syntenies joined by convergent evolution), we expect that the individual syntenies that compose them will be detected as well, and then we just need to remove the row corresponding to a chimeric synteny. However, depending on the nature of the data, one could very well consider other optimization criteria: for example, with genomic markers defined using virtual hybridization [39], or when considering duplicated genomic markers that represent ambiguous orthology relations, it would be natural to consider discarding columns of the matrix.

#### Orienting markers in CARs

In the present work, we do not orient markers in the set of CARs, unlike [12]. It is possible to adapt the present framework in order to consider marker orientations. It may be done by doubling every marker, as in [12], and adding an adjacency between the two copies with high weight, so that it is never removed during the optimization phase. Then the orientation will be inferred, with the possibility of remaining unresolved if the the two markers are involved in P-nodes of the PQ-tree: this means that the two orientations are equally possible.

#### Sensitivity to parameters

The first step of the method (detecting ancestral syntenies) captures more information as the resolution goes down (from 100 kb to 500 kb). So we are able to handle a resolution of 100 kb, but our best results are obtained for *max_gap* = 100 kb and *min_len* from 200 kb to 400 kb. This is probably because at higher resolution, the orthology and synteny signals are still perturbed by all kinds of duplications and repetitions, and at lower resolution, the coverage is too low to reconstruct reliable ancestors. At high resolution, in addition to the presence of many duplication and mobile elements, misassemblies and misplaced contigs may disturb the research for orthologies with the right positions (for example, the contigs may vary between two assembly versions of a genome, as we have seen for the bosTau3 and bosTau4 versions, leading to different results at high resolution). Apart from these considerations, the method is stable, in the sense that it recovers the same basic set of adjacencies for all choices of markers.

We also tested the sensitivity to branch lengths, and no results were altered by taking for example the branch lengths proposed by Ma *et al.* [12], based on an *a priori* amount of rearrangements that is expected in each branch. The method of Ma *et al.* [12], which we tested with the same parameter variability, was not as stable, due to the higher importance of its optimization step, which may give very different results with similar values.

## Material and Methods

### Computing Orthologous Markers from Whole Genome Alignments

We construct several datasets, by a unique method depending on two parameters, *max_gap* and *min_len*. This method, or very similar ones, are often used to construct synteny blocks from genomic alignments [40],[41],[59].

- We first downloaded the chained and netted pairwise alignments from the UCSC Genome Bioinformatics site [32] and the coordinates of all the alignments of the human genome (build hg18, March 2006 [60]) against respectively macaca (build rheMac2, January 2006 [61]), mouse (build mm9, July 2007 [62]), rat (build rn4, November 2004 [63]), cow (build bosTau4, October 2007 [64]), dog (build canFam2, May 2005 [65]), chicken (build galGal3, May 2006 [66]) and opossum (build monDom4, January 2006 [67]);
- For each set of alignments between the human genome and another genome, a graph is built, with vertices being the above alignments and edges joining two alignments if they have the same direction, and if they are not more distant than *max_gap*, a user-defined parameter (here 100 kb), in both genomes;
- Pairwise synteny blocks were defined as connected components of the above graphs that span a size of at least *min_len* of both genomes;
- The previous steps give a collection of pairwise breakpoints, with coordinates in the human genome. By considering all these breakpoints together, taking the union of those that intersect, we ended up with markers common to subsets of species, with their coordinates on the human genome and arrangements in all species, as sequences of markers (the chromosomes). We discarded the alignments which spanned less than 50kb of the human genome, and those which were at least 80% covered by segmental duplications. The coordinates of segmental duplications were also downloaded from the UCSC Genome Bioinformatics site [32].

### Ancestral Features: Gene Teams, Approximate Common Intervals, and Adjacencies

We first use the notion of “teams of markers” [24]. This notion relies on a parameter *δ*, a positive integer. In a genome, the *position* of a marker *m*, denoted by *p*(*m*), is its relative rank on the its chromosome. That is, the first marker on a chromosome has rank 1, the second has rank 2, and so on. Two markers *m*_1_ and *m*_2_ are said to be *close* to each other in a genome, for the parameter *δ*, if they lie on the same chromosome, and |*p*(*m*_1_)−*p*(*m*_2_)|≤*δ*. A subset of markers *M* is said to be a *team* for a genome if for any two markers *a*,*b* from *M*, there exists a sequence *S* = *a*,*a*_1_,…*a_k_*,*b* of markers from *M*, such that any two consecutive markers in *S* are close to each other. Given two genomes *X* and *Y*, a *team S* common to *X* and *Y* is a set of markers labels (a subset of Σ the alphabet of markers) that is a team in both genomes *X* and *Y*. Such a team *S* is *maximal* if no other team is common to *X* and *Y* and contains *S*. Maximal common intervals are maximal common teams for *δ* = 1. Maximal common teams can be computed efficiently thanks to an algorithm by Beal *et al.* [23] and a software described in [24]. We collect a set of teams, representing possible ancestral syntenies, by computing all maximal common teams of pairs of species which evolutionary path contains the wished ancestor.

In order to analyze the dataset of [12], due to less defined markers in the two outgroup genomes, we used maximal approximate common intervals defined as follows: a subset *M* of markers is an approximate common interval between two genomes if there exists a genome segment in each of the two genomes whose 80% of the gene content is equal to *S*. An approximate common interval *S* is *maximal* if no other approximate common intervals is common to *X* and *Y* and contains the two occurrences of *S* in *X* and *Y*.

As teams rely only on similarity in markers content, and do not involve any marker order constraints, we added to this set of ancestral syntenies the set of putative ancestral adjacencies, defined as pairs of markers that are consecutive in at least two genomes whose evolutionary path contains this ancestor and do not belong to a conflict. A conflict is defined as follows (Figure 7 in [12]): an adjacency {*i*, *j*} belongs to a conflict if, in the graph *G* whose vertices are the markers (*V*(*G*) = Σ) and the edges are the conserved adjacencies, either *i* or *j* has degree more than 2, or the edge {*i*, *j*} belongs to a cycle.

Each of these ancestral syntenies was weighted following the same principle as in [12]. Let *S* be a subset of Σ that represents a possible ancestral synteny. In any leaf *X* of the species tree, if *S* is a team in *X*, the weight of *S* in *X* is *w_X_*(*S*) = 1, otherwise, *w_X_*(*S*) = 0. Then, in any internal node *N* of *T* (other than the ancestral node *A*) having two children *R* and *L*, *w_N_*(*S*) is defined recursively by the formula
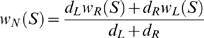
where *d_L_* and *d_R_* are respectively the length of the branch between *N* and *L* and *N* and *R*. The weight of *S* in *A* is then defined by
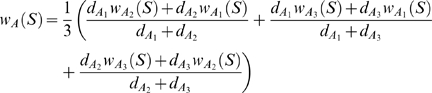
where *A*_1_, *A*_2_ and *A*_3_ are the three neighbors of the ancestral node *A* in *T*, and 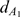, 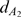 and 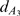 are the respective length of the branch between *A* and *A*_1_, *A* and *A*_2_ and *A* and *A*_3_.

### Construction of the Generalized PQ-Tree

Recall 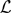 is the set of homologous markers, 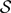 is the set of subsets of 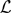 that represent possible ancestral syntenies and 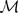 the corresponding 0/1 matrix.

We say that two elements *S_i_* and *S_j_* of 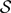 *overlap* if their intersection is not empty, but none is included in the other. Let 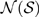 be the family of all subsets of 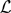 that do not overlap with any member of 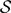; in other words, given *X* an element of 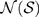, any *S_i_* of 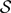 either contains all elements of *X* or contains no element of *X*. Among the subsets of 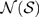, call *strong* the elements that do not overlap any other elements of 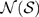. The *inclusion tree* of the strong elements of 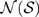, denoted 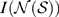, is a tree where each strong element of 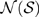 corresponds to a single node and the node corresponding to a strong subset *X* is an ancestor of the node corresponding to a strong subset *Y* if and only if *X* contains *Y* as a subset.

Given a node *N* of 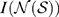, we associate to it the subset 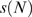 of the elements of 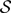 defined as all *S_i_*'s that are included in *N* but in none of its children. The PQ-tree 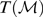 is defined from 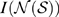 as follows: an internal node *N* such that *s*(*N*) = Ø is a P-node, while an internal node *N* such that *s*(*N*)≠Ø is a Q-node if *s*(*N*) can be partitioned by a partition refinement process [68] and a R-node otherwise. The construction of 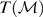 can be achieved in optimal *O*(*n*+*m*) time where 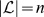 and 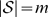, as described in [45].

### Algorithms for Clearing Ambiguities in Ancestral Syntenies

In the last step, we want to remove the minimal amount (in terms of weight) of ancestral syntenies from 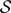 in order that the resulting matrix 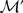 is C1P. This problem, which is known as the Consecutive Ones Submatrix Problem generalizes the Minimum Path Partition (or Path Cover) problem used in [12] and is known to be NP-hard [69] even for sparse matrices [70], which is the case of the matrices we obtain. However, using the structural information given by the PQ-tree 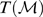, it is possible to design an efficient branch-and-bound algorithm.

More precisely, it follows immediately from the definition of 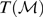 that ambiguous information that prevent a matrix 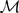 to be C1P can only be located in the submatrices defined by the subsets *s*(*N*) of 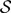 for the degenerate nodes of 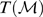. Hence each of these subsets of 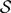 can be processed independently of the remaining of 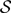. For such a subset, say 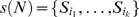, we first compute an upper bound on the maximum subset *S* of *s*(*N*) that defines a matrix that is C1P, using the same approach than in [12]: start with *S* = Ø and, for each element 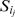 of *s*(*N*), taken in decreasing order of weight, if adding 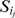 to *S* defines a matrix that is not C1P (which can be tested using the efficient algorithms described in [46],[68]), then discard it, else leave it in *S*. From that upper bound, using the same principle, we use a classical branch-and-bound algorithm that looks for a better subset of *s*(*N*) that defines a C1P matrix.

### Assessing the Stability of Adjacencies at Different Resolutions

Let an adjacency in an ancestral genome architecture be defined by two markers *X* and *Y* that are adjacent in a CAR of this ancestral genome, for a given resolution (say 100 kb). We say that it is conserved at a lower resolution (say 200 kb) if either the synteny blocks corresponding to *X* and *Y* in the human genome are both included in a single synteny block in the human genome at the lower resolution or if *X* and *Y* are contained in two blocks *X′* and *Y′* at the lower resolution level whose corresponding markers are adjacent in the ancestral genome inferred at this resolution. The adjacency is weakly conserved if the markers *X′* and *Y′* are not adjacent but present on the same CAR (weakly conserved adjacencies point at local rearrangements resulting from changing the resolution of the considered data). Otherwise, if the two markers *X′* and *Y′* are not on the same CAR, we say that the adjacency between *X* and *Y* is not conserved. Note that we do not consider this adjacency is not conserved if at least one of the two synteny blocks corresponding to *X* or *Y* is not included in a lower resolution synteny block.

## Acknowledgments

Part of this work was done while CC visited the LRI (Université Paris-Sud, Orsay, France) and LaBRI (Université Bordeaux I, Talence, France).
